# Supplementary material for: Development of a computer-aided design software for the quantitative evaluation of aesthetic damage
Source: PLoS One. 2019 Dec 18;14(12):e0226322. doi: 10.1371/journal.pone.0226322 (PMC6919621; doi:10.1371/journal.pone.0226322)
Supplement: S1 Code site — 1. (ZIP) [file pone.0226322.s001.zip › [EESC_jr][Projeto]Relatorio_07_21/reports/templates_zip/formwizard-master.zip!/formwizard-master/examples/validate.html]

{"invalidcharacters" : false, "invalidcharactersmessage" : "The first name contained invalid characters","emailtaken" : true, "emailerrormessage" : "The email is unfortunately already taken. Please contact our support for details."}
